# Supplementary material for: Synthesis, Structure, Spectral-Luminescent Properties, and Biological Activity of Chlorine-Substituted N-[2-(Phenyliminomethyl)phenyl]-4-methylbenzenesulfamide and Their Zinc(II) Complexes
Source: Int J Mol Sci. 2022 Dec 3;23(23):15259. doi: 10.3390/ijms232315259 (PMC9741117; doi:10.3390/ijms232315259)
Supplement: Supplementary file 1 [file ijms-23-15259-s001.zip › ijms-2041272-supplementary.pdf]

Synthesis, Structure, Spectral-Luminescent Properties, and Biological Activity of Chlorine-Substituted *N*-[2-(Phenyliminomethyl)phenyl]-4-methylbenzenesulfamide and Their Zinc(II) Complexes

Anatolii S. Burlov [1], Valery G. Vlasenko [2], Maxim S. Milutka [1], Yurii V. Koshchienko [1], Nadezhda I. Makarova [1], Vladimir A. Lazarenko [3], Alexander L. Trigub [3], Alexandra A. Kolodina [1], Alexander A. Zubenko [4], Anatoly V. Metelitsa [1], Dmitrii A. Garnovskii [5], Alexey N. Gusev [6,*] and Wolfgang Linert [7]

[1] Institute of Physical and Organic Chemistry, Southern Federal University, 344090 Rostov-on-Don, Russia; anatoly.burlov@yandex.ru (A.S.B.); milutka.maxim@yandex.ru (M.S.M.); yukoshch@yandex.ru (Y.V.K.); nimakarova@sfedu.ru (N.I.M.); akolodina@sfedu.ru (A.A.K.); avmetelitsa@sfedu.ru (A.V.M.)

[2] Institute of Physics, Southern Federal University, 344090 Rostov-on-Don, Russia; vgvlasenko@sfedu.ru

[3] National Research Centre "Kurchatov Institute", 123182 Moscow, Russia; vladimir.a.lazarenko@gmail.com (V.A.L.); alexander.trigub@gmail.com (A.L.T.)

[4] North-Caucasian Zonal Scientific Research Veterinary Institute, Branch of the Federal State Budget Scientific Institution "Federal Rostov Agricultural Research Centre", 346421 Novocherkassk, Russia; alexsandrzubenko@yandex.ru

[5] Southern Scientific Center of Russian Academy of Sciences, 344006 Rostov-on-Don, Russia; ayueliseeva@mail.ru

[6] General Chemistry Department, Crimean Federal University V.I. Vernadsky, 295007 Simferopol, Russia

[7] Institute of Applied Physics, Vienna University of Technology, Wiedner Hauptstraße 8-10, 1040 Vienna, Austria; wolfgang.linert@tuwien.ac.at

* Correspondence: galex0330@gmail.com; Tel.: +7-978-723-4813

Table S1. Crystal data and structure refinement for **2a** and **2d**.

CCDC	2203056	2203057
Identification code	**2a**	**2d**
Empirical formula	$C_{40}H_{32}Cl2N_4O_4S_2Zn$	$C_{40}H_{28}Cl_6N_4O_4S_2Zn$
Formula weight	833.08	970.87
Temperature	100(2) K	100(2) K
Wavelength	0.7454 Å	0.71073 Å
Crystal system	Monoclinic	Monoclinic
Space group	*C*2/c	*C*2/c
Unit cell dimensions	a = 18.568(4) Å	a = 14.7467(3) Å
	b = 20.748(4) Å	b = 20.4680(4) Å
	c = 10.689(2) Å	c = 13.6156(3) Å
	α = 90°	α = 90°
	β = 114.69(3)°	β = 100.7570(10)°
	γ = 90°	γ = 90°
Volume	3741.4 (15) Å^3	4037.45(15) Å^3
Z	4	4
Density (calculated)	1.4797 Mg/m^3	1.597 Mg/m^3
Absorption coefficient	1.08 mm^{-1}	1.157 mm^{-1}
F(000)	1712	1968
Crystal size	0.3 × 0.1 × 0.05 mm^3	0.18 x 0.09 x 0.09 mm^3
Theta range for data collection	1.6 to 31.0°	2.118 to 32.697°
Index ranges	-25<=h<=24, -28<=k<=28, -14<=l<=14	-22<=h<=22, -31<=k<=30, -20<=l<=20
Reflections collected	19362	46971
Independent reflections	4459	7402 [R(int) = 0.0485]
Completeness to theta = 25.242°	99.9 %	99.9 %
Absorption correction	Semi-empirical from equivalents	Semi-empirical from equivalents
Max. and min. transmission	1.000 and 0.721	0.888 and 0.800
Refinement method	Full-matrix least-squares on F^2	Full-matrix least-squares on F^2
Data / restraints / parameters	4869 / 0 / 241	7402 / 0 / 259
Goodness-of-fit on F^2	1.09	1.028
R indices (all data)	R_{int} = 0.0596, R1 = 0.389, wR2 = 0.0989	R_{int} = 0.0485, R1 = 0.0463, wR2 = 0.0754
Extinction coefficient	n/a	n/a
Largest diff. peak and hole	0.45 and -0.86 e, Å^{-3}	0.477 and -0.425 e, Å^{-3}

Table S2. Atomic coordinates (x 10^4) and equivalent isotropic displacement parameters (Å^2 x 10^3) for **2a**. U(eq) is defined as one third of the trace of the orthogonalized U^{ij} tensor.

Atom	*x*	*y*	*z*	U_{iso}*/U_{eq}
Zn1	0.500000	0.22198 (2)	0.250000	0.02002 (8)
S1	0.47203 (2)	0.30795 (2)	0.44491 (4)	0.01994 (9)
Cl1	0.63733 (3)	0.01791 (3)	-0.14208 (6)	0.04547 (14)
O1	0.42074 (7)	0.31838 (6)	0.30061 (12)	0.0231 (2)
O2	0.43308 (7)	0.29498 (6)	0.53395 (12)	0.0240 (2)
N2	0.60157 (8)	0.16902 (6)	0.30037 (14)	0.0216 (3)
N1	0.53148 (8)	0.25248 (6)	0.44080 (14)	0.0207 (3)
C13	0.59705 (9)	0.23392 (7)	0.55956 (17)	0.0211 (3)
C8	0.65972 (9)	0.19879 (7)	0.54675 (17)	0.0224 (3)
C7	0.65913 (9)	0.17094 (8)	0.42223 (17)	0.0233 (3)
H7	0.706311	0.151890	0.430833	0.028*
C4	0.61319 (9)	0.13530 (7)	0.19374 (17)	0.0224 (3)
C14	0.53196 (9)	0.37722 (7)	0.51164 (17)	0.0223 (3)
C11	0.67372 (10)	0.23400 (9)	0.80845 (18)	0.0270 (3)
H11	0.677172	0.244143	0.895524	0.032*
C12	0.60542 (10)	0.24953 (8)	0.69290 (17)	0.0250 (3)
H12	0.564333	0.270718	0.704002	0.030*
C9	0.72906 (10)	0.18592 (8)	0.66641 (18)	0.0263 (3)
H9	0.770877	0.164794	0.657593	0.032*
C19	0.51859 (10)	0.41530 (8)	0.60619 (18)	0.0264 (3)
H19	0.478076	0.405187	0.632433	0.032*
C5	0.68596 (10)	0.13276 (9)	0.18456 (18)	0.0272 (3)
H5	0.729340	0.154932	0.248114	0.033*
C15	0.59143 (10)	0.39245 (8)	0.46996 (19)	0.0265 (3)
H15	0.599437	0.367164	0.405050	0.032*
C10	0.73707 (10)	0.20348 (9)	0.79570 (18)	0.0284 (3)
H10	0.783785	0.195131	0.872867	0.034*
C17	0.62732 (10)	0.48461 (8)	0.62368 (18)	0.0260 (3)
C3	0.54763 (10)	0.10435 (8)	0.09382 (18)	0.0260 (3)
H3	0.498541	0.107418	0.097343	0.031*
C18	0.56649 (10)	0.46890 (8)	0.66155 (19)	0.0288 (3)
H18	0.557600	0.494613	0.724966	0.035*
C16	0.63882 (10)	0.44605 (8)	0.52668 (19)	0.0279 (3)
H16	0.678896	0.456380	0.499477	0.034*
C6	0.69375 (11)	0.09728 (9)	0.0810 (2)	0.0320 (4)
H6	0.742355	0.095055	0.075482	0.038*
C2	0.55475 (11)	0.06916 (9)	-0.01039 (19)	0.0295 (4)
H2	0.510925	0.048574	-0.076675	0.035*
C1	0.62824 (11)	0.06509 (9)	-0.01436 (19)	0.0306 (4)
C20	0.67908 (11)	0.54212 (9)	0.6857 (2)	0.0324 (4)
H20A	0.651980	0.571477	0.720729	0.049*
H20B	0.690790	0.563334	0.616570	0.049*
H20C	0.727566	0.528311	0.759524	0.049*

Table S3. Bond lengths [Å] and angles [°] for **2a**.

Zn1—N2	2.0508 (14)	C4—C5	1.396 (2)
Zn1—N2^{i}	2.0507 (14)	C4—C3	1.396 (2)
Zn1—N1^{i}	1.9763 (14)	C14—C19	1.384 (2)
Zn1—N1	1.9763 (14)	C14—C15	1.389 (2)
S1—O1	1.4511 (13)	C11—C12	1.389 (2)
S1—O2	1.4410 (13)	C11—C10	1.392 (2)
S1—N1	1.6078 (14)	C9—C10	1.376 (3)
S1—C14	1.7747 (16)	C19—C18	1.392 (2)
Cl1—C1	1.7444 (19)	C5—C6	1.386 (3)
N2—C7	1.295 (2)	C15—C16	1.390 (2)
N2—C4	1.429 (2)	C17—C18	1.388 (3)
N1—C13	1.397 (2)	C17—C16	1.395 (2)
C13—C8	1.427 (2)	C17—C20	1.502 (2)
C13—C12	1.406 (2)	C3—C2	1.384 (2)
C8—C7	1.447 (2)	C6—C1	1.389 (3)
C8—C9	1.410 (2)	C2—C1	1.385 (3)
N2^{i}—Zn1—N2	115.21 (8)	C5—C4—N2	123.21 (14)
N1—Zn1—N2^{i}	106.60 (6)	C5—C4—C3	119.29 (16)
N1^{i}—Zn1—N2	106.60 (6)	C3—C4—N2	117.50 (15)
N1^{i}—Zn1—N2^{i}	93.30 (6)	C19—C14—S1	118.82 (13)
N1—Zn1—N2	93.30 (6)	C19—C14—C15	120.89 (15)
N1—Zn1—N1^{i}	142.64 (8)	C15—C14—S1	120.29 (13)
O1—S1—N1	103.35 (7)	C12—C11—C10	120.91 (17)
O1—S1—C14	109.23 (8)	C11—C12—C13	121.43 (16)
O2—S1—O1	116.25 (7)	C10—C9—C8	122.38 (16)
O2—S1—N1	115.09 (7)	C14—C19—C18	119.25 (16)
O2—S1—C14	106.81 (8)	C6—C5—C4	120.20 (16)
N1—S1—C14	105.57 (7)	C14—C15—C16	119.03 (16)
C7—N2—Zn1	121.98 (12)	C9—C10—C11	118.52 (15)
C7—N2—C4	118.77 (14)	C18—C17—C16	118.55 (15)
C4—N2—Zn1	118.80 (10)	C18—C17—C20	120.42 (16)
S1—N1—Zn1	110.30 (7)	C16—C17—C20	121.03 (16)
C13—N1—Zn1	128.27 (11)	C2—C3—C4	120.84 (16)
C13—N1—S1	121.35 (11)	C17—C18—C19	121.11 (16)
N1—C13—C8	119.32 (15)	C15—C16—C17	121.16 (16)
N1—C13—C12	122.91 (15)	C5—C6—C1	119.34 (17)
C12—C13—C8	117.75 (14)	C3—C2—C1	118.90 (16)
C13—C8—C7	126.79 (14)	C6—C1—Cl1	120.20 (15)
C9—C8—C13	118.80 (15)	C2—C1—Cl1	118.46 (14)
C9—C8—C7	114.33 (14)	C2—C1—C6	121.33 (17)
N2—C7—C8	128.57 (15)		

Symmetry code: (i) -*x*+1, *y*, -*z*+1/2.

Table S4. Anisotropic displacement parameters (Å^2 x 10^3) for **2a**. The anisotropic displacement factor exponent takes the form: $-2\pi^2$[h^2 $a^{*2}U^{11}$ + ... + 2 h k a* b* U^{12}]

	U^{11}	U^{22}	U^{33}	U^{12}	U^{13}	U^{23}
Zn1	0.01965 (13)	0.01809 (13)	0.01991 (13)	0.000	0.00588 (10)	0.000
S1	0.01884 (16)	0.01908 (17)	0.02066 (18)	0.00102 (12)	0.00703 (14)	-0.00033 (12)
Cl1	0.0437 (3)	0.0542 (3)	0.0416 (3)	0.0015 (2)	0.0209 (2)	-0.0204 (2)
O1	0.0214 (5)	0.0236 (5)	0.0218 (5)	0.0029 (4)	0.0064 (4)	0.0008 (4)
O2	0.0220 (5)	0.0260 (5)	0.0248 (6)	-0.0003 (4)	0.0106 (4)	0.0002 (4)
N2	0.0219 (6)	0.0178 (6)	0.0236 (6)	0.0016 (4)	0.0080 (5)	0.0003 (5)
N1	0.0211 (6)	0.0203 (6)	0.0190 (6)	0.0011 (4)	0.0067 (5)	-0.0013 (5)
C13	0.0201 (6)	0.0176 (6)	0.0232 (7)	0.0004 (5)	0.0067 (6)	0.0003 (5)
C8	0.0222 (7)	0.0197 (7)	0.0229 (7)	0.0013 (5)	0.0070 (6)	0.0001 (5)
C7	0.0223 (7)	0.0202 (7)	0.0258 (8)	0.0023 (5)	0.0085 (6)	0.0001 (6)
C4	0.0246 (7)	0.0186 (6)	0.0227 (7)	0.0024 (5)	0.0086 (6)	0.0010 (5)
C14	0.0223 (7)	0.0195 (7)	0.0233 (7)	0.0013 (5)	0.0078 (6)	-0.0004 (5)
C11	0.0267 (8)	0.0286 (8)	0.0221 (7)	0.0033 (6)	0.0067 (6)	-0.0016 (6)
C12	0.0241 (7)	0.0250 (7)	0.0241 (8)	0.0025 (6)	0.0083 (6)	-0.0004 (6)
C9	0.0226 (7)	0.0250 (7)	0.0269 (8)	0.0050 (6)	0.0061 (6)	-0.0006 (6)
C19	0.0255 (7)	0.0257 (8)	0.0292 (8)	-0.0020 (6)	0.0125 (6)	-0.0050 (6)
C5	0.0252 (7)	0.0283 (8)	0.0275 (8)	-0.0001 (6)	0.0104 (6)	-0.0028 (6)
C15	0.0300 (8)	0.0222 (7)	0.0301 (8)	-0.0019 (6)	0.0154 (7)	-0.0043 (6)
C10	0.0252 (7)	0.0282 (8)	0.0246 (8)	0.0047 (6)	0.0034 (6)	-0.0012 (6)
C17	0.0261 (7)	0.0198 (7)	0.0297 (8)	0.0006 (5)	0.0092 (6)	-0.0019 (6)
C3	0.0242 (7)	0.0239 (7)	0.0290 (8)	0.0000 (6)	0.0103 (6)	-0.0023 (6)
C18	0.0296 (8)	0.0259 (8)	0.0320 (9)	-0.0021 (6)	0.0139 (7)	-0.0081 (6)
C16	0.0291 (8)	0.0236 (8)	0.0341 (9)	-0.0034 (6)	0.0161 (7)	-0.0029 (6)
C6	0.0277 (8)	0.0366 (9)	0.0336 (9)	0.0003 (7)	0.0145 (7)	-0.0058 (7)
C2	0.0307 (8)	0.0270 (8)	0.0297 (8)	-0.0019 (6)	0.0116 (7)	-0.0055 (6)
C1	0.0353 (9)	0.0303 (8)	0.0282 (8)	0.0020 (7)	0.0151 (7)	-0.0062 (7)
C20	0.0317 (8)	0.0241 (8)	0.0428 (10)	-0.0033 (6)	0.0170 (8)	-0.0077 (7)

Table S5. Atomic coordinates (x 10^4) and equivalent isotropic displacement parameters (Å^2 x 10^3) for **2d**. U(eq) is defined as one third of the trace of the orthogonalized U^{ij} tensor.

Atom	x	y	z	U(eq)
Zn(1)	5000	5313(1)	2500	11(1)
Cl(1)	9403(1)	5342(1)	739(1)	26(1)
Cl(2)	5448(1)	2173(1)	1176(1)	26(1)
Cl(3)	3286(1)	2197(1)	957(1)	29(1)
S(1)	6273(1)	6238(1)	3728(1)	12(1)
O(1)	5389(1)	6147(1)	4031(1)	16(1)
O(2)	7074(1)	6174(1)	4516(1)	17(1)
N(1)	6243(1)	5719(1)	2831(1)	13(1)
N(2)	5415(1)	4662(1)	1537(1)	13(1)
C(1)	6994(1)	5629(1)	2356(1)	12(1)
C(2)	6919(1)	5186(1)	1539(1)	12(1)
C(3)	7671(1)	5111(1)	1038(1)	15(1)
C(4)	8476(1)	5450(1)	1345(1)	16(1)
C(5)	8571(1)	5864(1)	2162(1)	17(1)
C(6)	7841(1)	5954(1)	2652(1)	16(1)
C(7)	6183(1)	4723(1)	1214(1)	13(1)
C(8)	6313(1)	7027(1)	3209(1)	14(1)
C(9)	6532(1)	7552(1)	3860(1)	19(1)
C(10)	6505(1)	8181(1)	3474(1)	23(1)
C(11)	6262(1)	8295(1)	2444(1)	21(1)
C(12)	6057(1)	7761(1)	1815(1)	18(1)
C(13)	6079(1)	7127(1)	2185(1)	16(1)
C(14)	6214(1)	8980(1)	2033(1)	32(1)
C(15)	4904(1)	4074(1)	1293(1)	13(1)
C(16)	5363(1)	3479(1)	1301(1)	15(1)
C(17)	4860(1)	2904(1)	1158(1)	17(1)
C(18)	3903(1)	2917(1)	1031(1)	18(1)
C(19)	3446(1)	3512(1)	1005(1)	20(1)
C(20)	3944(1)	4090(1)	1140(1)	17(1)

Table S6. Bond lengths [Å] and angles [°] for **2d**.

Zn(1)-N(1)#1	1.9867(12)	C(7)-H(7)	0.9500
Zn(1)-N(1)	1.9867(12)	C(8)-C(13)	1.3880(19)
Zn(1)-N(2)#1	2.0407(11)	C(8)-C(9)	1.3915(19)
Zn(1)-N(2)	2.0408(11)	C(9)-C(10)	1.390(2)
Cl(1)-C(4)	1.7381(14)	C(9)-H(9)	0.9500
Cl(2)-C(17)	1.7284(15)	C(10)-C(11)	1.400(2)
Cl(3)-C(18)	1.7241(14)	C(10)-H(10)	0.9500
S(1)-O(2)	1.4450(11)	C(11)-C(12)	1.387(2)
S(1)-O(1)	1.4500(10)	C(11)-C(14)	1.506(2)
S(1)-N(1)	1.6126(11)	C(12)-C(13)	1.390(2)
S(1)-C(8)	1.7676(14)	C(12)-H(12)	0.9500
N(1)-C(1)	1.3956(17)	C(13)-H(13)	0.9500
N(2)-C(7)	1.2954(17)	C(14)-H(14A)	0.9800
N(2)-C(15)	1.4255(17)	C(14)-H(14B)	0.9800
C(1)-C(6)	1.4064(19)	C(14)-H(14C)	0.9800
C(1)-C(2)	1.4223(18)	C(15)-C(20)	1.393(2)
C(2)-C(3)	1.4158(18)	C(15)-C(16)	1.3939(19)
C(2)-C(7)	1.4473(19)	C(16)-C(17)	1.384(2)
C(3)-C(4)	1.371(2)	C(16)-H(16)	0.9500
C(3)-H(3)	0.9500	C(17)-C(18)	1.389(2)
C(4)-C(5)	1.384(2)	C(18)-C(19)	1.389(2)
C(5)-C(6)	1.3810(19)	C(19)-C(20)	1.387(2)
C(5)-H(5)	0.9500	C(19)-H(19)	0.9500
C(6)-H(6)	0.9500	C(20)-H(20)	0.9500
N(1)#1-Zn(1)-N(1)	130.47(7)	S(1)-N(1)-Zn(1)	109.97(6)
N(1)#1-Zn(1)-N(2)#1	92.57(5)	C(7)-N(2)-C(15)	117.31(11)
N(1)-Zn(1)-N(2)#1	120.12(5)	C(7)-N(2)-Zn(1)	122.80(9)
N(1)#1-Zn(1)-N(2)	120.12(5)	C(15)-N(2)-Zn(1)	119.46(9)
N(1)-Zn(1)-N(2)	92.57(5)	N(1)-C(1)-C(6)	122.89(12)
N(2)#1-Zn(1)-N(2)	98.56(6)	N(1)-C(1)-C(2)	119.63(12)
O(2)-S(1)-O(1)	115.55(6)	C(6)-C(1)-C(2)	117.49(12)
O(2)-S(1)-N(1)	113.91(6)	C(3)-C(2)-C(1)	119.54(12)
O(1)-S(1)-N(1)	103.38(6)	C(3)-C(2)-C(7)	113.03(12)
O(2)-S(1)-C(8)	107.21(6)	C(1)-C(2)-C(7)	126.95(12)
O(1)-S(1)-C(8)	109.21(6)	C(4)-C(3)-C(2)	120.60(13)
N(1)-S(1)-C(8)	107.25(6)	C(4)-C(3)-H(3)	119.7
C(1)-N(1)-S(1)	121.78(9)	C(2)-C(3)-H(3)	119.7
C(1)-N(1)-Zn(1)	128.13(9)	C(3)-C(4)-C(5)	120.44(13)

C(3)-C(4)-Cl(1)	120.22(11)	C(8)-C(13)-H(13)	120.4
C(5)-C(4)-Cl(1)	119.30(11)	C(12)-C(13)-H(13)	120.4
C(6)-C(5)-C(4)	119.99(13)	C(11)-C(14)-H(14A)	109.5
C(6)-C(5)-H(5)	120.0	C(11)-C(14)-H(14B)	109.5
C(4)-C(5)-H(5)	120.0	H(14A)-C(14)-H(14B)	109.5
C(5)-C(6)-C(1)	121.88(13)	C(11)-C(14)-H(14C)	109.5
C(5)-C(6)-H(6)	119.1	H(14A)-C(14)-H(14C)	109.5
C(1)-C(6)-H(6)	119.1	H(14B)-C(14)-H(14C)	109.5
N(2)-C(7)-C(2)	127.75(12)	C(20)-C(15)-C(16)	120.00(13)
N(2)-C(7)-H(7)	116.1	C(20)-C(15)-N(2)	119.60(12)
C(2)-C(7)-H(7)	116.1	C(16)-C(15)-N(2)	120.12(12)
C(13)-C(8)-C(9)	120.66(13)	C(17)-C(16)-C(15)	119.67(13)
C(13)-C(8)-S(1)	121.08(10)	C(17)-C(16)-H(16)	120.2
C(9)-C(8)-S(1)	118.14(11)	C(15)-C(16)-H(16)	120.2
C(10)-C(9)-C(8)	119.22(13)	C(16)-C(17)-C(18)	120.49(13)
C(10)-C(9)-H(9)	120.4	C(16)-C(17)-Cl(2)	118.66(12)
C(8)-C(9)-H(9)	120.4	C(18)-C(17)-Cl(2)	120.83(11)
C(9)-C(10)-C(11)	121.10(14)	C(19)-C(18)-C(17)	119.72(13)
C(9)-C(10)-H(10)	119.5	C(19)-C(18)-Cl(3)	119.91(12)
C(11)-C(10)-H(10)	119.5	C(17)-C(18)-Cl(3)	120.31(11)
C(12)-C(11)-C(10)	118.28(14)	C(20)-C(19)-C(18)	120.17(14)
C(12)-C(11)-C(14)	120.93(14)	C(20)-C(19)-H(19)	119.9
C(10)-C(11)-C(14)	120.78(14)	C(18)-C(19)-H(19)	119.9
C(11)-C(12)-C(13)	121.57(13)	C(19)-C(20)-C(15)	119.90(14)
C(11)-C(12)-H(12)	119.2	C(19)-C(20)-H(20)	120.1
C(13)-C(12)-H(12)	119.2	C(15)-C(20)-H(20)	120.1
C(8)-C(13)-C(12)	119.16(13)		

Symmetry transformations used to generate equivalent atoms:

#1 -x+1, y, -z+1/2

Table S7. Anisotropic displacement parameters ($Å^2 \times 10^3$) for **2d**. The anisotropic displacement factor exponent takes the form: $-2\pi^2[h^2 a^{*2}U^{11} + ... + 2 h k a^* b^* U^{12}]$

Atom	U^{11}	U^{22}	U^{33}	U^{23}	U^{13}	U^{12}
Zn(1)	12(1)	11(1)	12(1)	0	3(1)	0
Cl(1)	18(1)	32(1)	30(1)	-6(1)	14(1)	-2(1)
Cl(2)	34(1)	13(1)	30(1)	1(1)	6(1)	3(1)
Cl(3)	31(1)	18(1)	34(1)	4(1)	-3(1)	-11(1)
S(1)	15(1)	13(1)	9(1)	-1(1)	2(1)	0(1)
O(1)	18(1)	18(1)	13(1)	-1(1)	6(1)	-1(1)
O(2)	19(1)	18(1)	12(1)	1(1)	-1(1)	-1(1)
N(1)	13(1)	13(1)	12(1)	-2(1)	4(1)	-1(1)
N(2)	14(1)	12(1)	11(1)	-1(1)	2(1)	-1(1)
C(1)	14(1)	12(1)	12(1)	1(1)	3(1)	1(1)
C(2)	14(1)	12(1)	12(1)	0(1)	4(1)	-1(1)
C(3)	18(1)	15(1)	14(1)	0(1)	6(1)	1(1)
C(4)	14(1)	17(1)	19(1)	1(1)	8(1)	0(1)
C(5)	13(1)	17(1)	20(1)	-1(1)	4(1)	-2(1)
C(6)	16(1)	14(1)	17(1)	-3(1)	4(1)	-2(1)
C(7)	16(1)	13(1)	12(1)	-1(1)	3(1)	0(1)
C(8)	15(1)	12(1)	13(1)	-1(1)	2(1)	0(1)
C(9)	24(1)	17(1)	15(1)	-4(1)	-1(1)	0(1)
C(10)	32(1)	14(1)	22(1)	-5(1)	-2(1)	-2(1)
C(11)	24(1)	14(1)	23(1)	1(1)	1(1)	-1(1)
C(12)	24(1)	16(1)	15(1)	1(1)	1(1)	-1(1)
C(13)	20(1)	14(1)	12(1)	-1(1)	2(1)	-2(1)
C(14)	45(1)	15(1)	32(1)	3(1)	-1(1)	-2(1)
C(15)	17(1)	12(1)	10(1)	-1(1)	4(1)	-2(1)
C(16)	17(1)	16(1)	13(1)	0(1)	4(1)	0(1)
C(17)	25(1)	12(1)	13(1)	1(1)	3(1)	1(1)
C(18)	22(1)	15(1)	16(1)	1(1)	1(1)	-6(1)
C(19)	16(1)	21(1)	22(1)	-1(1)	1(1)	-4(1)
C(20)	16(1)	16(1)	17(1)	-1(1)	1(1)	-1(1)

Table S8. Hydrogen coordinates (x 10^4) and isotropic displacement parameters (Å^2 x 10^3) for **2d**.

Atom	x	y	z	U(iso)
H(3)	7618	4823	483	18
H(5)	9138	6086	2385	20
H(6)	7913	6245	3206	19
H(7)	6275	4427	704	16
H(9)	6698	7480	4560	23
H(10)	6654	8541	3915	28
H(12)	5898	7830	1114	22
H(13)	5935	6767	1743	19
H(14A)	5963	8969	1314	48
H(14B)	6835	9170	2146	48
H(14C)	5813	9246	2371	48
H(16)	6018	3467	1404	18
H(19)	2791	3522	894	24
H(20)	3630	4496	1128	20

Table S9. Torsion angles [°] for **2d**.

O(2)-S(1)-N(1)-C(1)	-50.82(12)	O(2)-S(1)-C(8)-C(9)	-44.35(13)
O(1)-S(1)-N(1)-C(1)	-177.01(10)	O(1)-S(1)-C(8)-C(9)	81.53(13)
C(8)-S(1)-N(1)-C(1)	67.65(12)	N(1)-S(1)-C(8)-C(9)	-167.07(11)
O(2)-S(1)-N(1)-Zn(1)	132.79(6)	C(13)-C(8)-C(9)-C(10)	0.7(2)
O(1)-S(1)-N(1)-Zn(1)	6.60(8)	S(1)-C(8)-C(9)-C(10)	-175.58(12)
C(8)-S(1)-N(1)-Zn(1)	-108.75(7)	C(8)-C(9)-C(10)-C(11)	0.0(3)
S(1)-N(1)-C(1)-C(6)	3.30(18)	C(9)-C(10)-C(11)-C(12)	-0.6(3)
Zn(1)-N(1)-C(1)-C(6)	178.99(10)	C(9)-C(10)-C(11)-C(14)	178.63(17)
S(1)-N(1)-C(1)-C(2)	-177.23(10)	C(10)-C(11)-C(12)-C(13)	0.7(2)
Zn(1)-N(1)-C(1)-C(2)	-1.54(18)	C(14)-C(11)-C(12)-C(13)	-178.56(16)
N(1)-C(1)-C(2)-C(3)	177.99(12)	C(9)-C(8)-C(13)-C(12)	-0.6(2)
C(6)-C(1)-C(2)-C(3)	-2.51(19)	S(1)-C(8)-C(13)-C(12)	175.53(12)
N(1)-C(1)-C(2)-C(7)	-10.6(2)	C(11)-C(12)-C(13)-C(8)	-0.1(2)
C(6)-C(1)-C(2)-C(7)	168.94(13)	C(7)-N(2)-C(15)-C(20)	-147.27(13)
C(1)-C(2)-C(3)-C(4)	1.4(2)	Zn(1)-N(2)-C(15)-C(20)	40.02(16)
C(7)-C(2)-C(3)-C(4)	-171.21(13)	C(7)-N(2)-C(15)-C(16)	38.82(18)
C(2)-C(3)-C(4)-C(5)	1.0(2)	Zn(1)-N(2)-C(15)-C(16)	-133.90(11)
C(2)-C(3)-C(4)-Cl(1)	178.80(11)	C(20)-C(15)-C(16)-C(17)	-0.1(2)
C(3)-C(4)-C(5)-C(6)	-2.1(2)	N(2)-C(15)-C(16)-C(17)	173.79(12)
Cl(1)-C(4)-C(5)-C(6)	-179.97(11)	C(15)-C(16)-C(17)-C(18)	-1.6(2)
C(4)-C(5)-C(6)-C(1)	0.9(2)	C(15)-C(16)-C(17)-Cl(2)	-179.78(10)
N(1)-C(1)-C(6)-C(5)	-179.11(13)	C(16)-C(17)-C(18)-C(19)	2.8(2)
C(2)-C(1)-C(6)-C(5)	1.4(2)	Cl(2)-C(17)-C(18)-C(19)	-179.07(11)
C(15)-N(2)-C(7)-C(2)	-164.58(13)	C(16)-C(17)-C(18)-Cl(3)	-174.59(11)
Zn(1)-N(2)-C(7)-C(2)	7.9(2)	Cl(2)-C(17)-C(18)-Cl(3)	3.58(18)
C(3)-C(2)-C(7)-N(2)	179.10(13)	C(17)-C(18)-C(19)-C(20)	-2.3(2)
C(1)-C(2)-C(7)-N(2)	7.2(2)	Cl(3)-C(18)-C(19)-C(20)	175.09(12)
O(2)-S(1)-C(8)-C(13)	139.42(12)	C(18)-C(19)-C(20)-C(15)	0.6(2)
O(1)-S(1)-C(8)-C(13)	-94.70(13)	C(16)-C(15)-C(20)-C(19)	0.6(2)
N(1)-S(1)-C(8)-C(13)	16.70(14)	N(2)-C(15)-C(20)-C(19)	-173.34(13)

Symmetry transformations used to generate equivalent atoms:

#1 -x+1, y, -z+1/2

Elemental analysis of the azomethines **1a-e** and complexes **2a-e**

БЛАНК ЗАКАЗА НА ПРОВЕДЕНИЕ ИССЛЕДОВАНИЯ СТРУКТУРЫ СОЕДИНЕНИЙ

1a

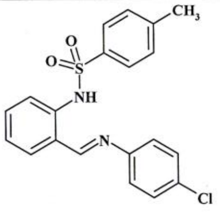

Дата получения заказа<br>28.02.2020	Дата проведения анализа<br>06.03.2020	Масса образца, мг
Шифр<br>**М-149**	Мол. масса	Брутто-формула<br>$C_{20}H_{17}ClN_2O_2S$
Физ. состояние<br>***порошок***	Т. пл. (Т. кип.)<br>133-134˚C	Устойчивость при хранении<br>***устойчив***

Синтетик (лаборатория, факультет, подпись, телефон)<br>**Милутка М.**С. Отдел координационных соединений НИИФОХ ЮФУ<br>+7(863) 297-51-89	Зав. лаб. (рук. гр.) Фамилия, подпись<br>**А.С. Бурлов,**
Оператор (Фамилия, подпись)	Комментарии оператора<br>***нет***

ЯМР	ИК	УФ	Элементный анализ				
Исследуемое ядро			Элемент	**C**	**H**	**N**	Др.
Растворитель			Вычислено, %	62.41	4.45	7.28	
Стандарт			Найдено, %	62.40<br>62.48	4.44<br>4.52	7.27<br>7.35	
			Ср.	62.44	4.48	7.31	

БЛАНК ЗАКАЗА НА ПРОВЕДЕНИЕ ИССЛЕДОВАНИЯ СТРУКТУРЫ СОЕДИНЕНИЙ

1b

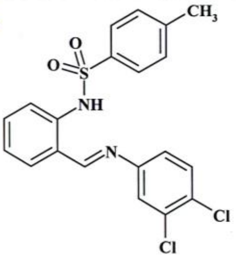

Дата получения заказа<br>02.03.2020	Дата проведения анализа<br>09.03.2020	Масса образца, мг
Шифр<br>**М-150**	Мол. масса	Брутто-формула<br>$C_{20}H_{16}Cl_2N_2O_2S$
Физ. состояние<br>***порошок***	Т. пл. (Т. кип.)<br>139-140˚C	Устойчивость при хранении<br>***устойчив***

Синтетик (лаборатория, факультет, подпись, телефон)<br>**Милутка М.**С. Отдел координационных соединений НИИФОХ ЮФУ<br>+7(863) 297-51-89	Зав. лаб. (рук. гр.) Фамилия, подпись<br>**А.С. Бурлов,**
Оператор (Фамилия, подпись)<br>Этметиенко	Комментарии оператора<br>***нет***

ЯМР	ИК	УФ	Элементный анализ				
Исследуемое ядро			Элемент	**C**	**H**	**N**	Др.
Растворитель			Вычислено, %	57.29	3.85`	6.68	
Стандарт			Найдено, %	57,21<br>57,29	3,75<br>3,87	6,60<br>6,68	
			Ср.	57,25	3,81	6,64	

БЛАНК ЗАКАЗА НА ПРОВЕДЕНИЕ ИССЛЕДОВАНИЯ СТРУКТУРЫ СОЕДИНЕНИЙ

1c

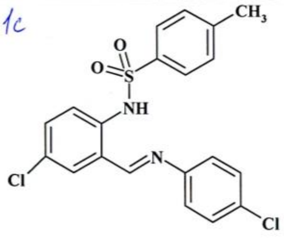

	Дата получения заказа<br>05.03.2020	Дата проведения анализа<br>12.03.2020	Масса образца, мг
	Шифр<br>**М-146**	Мол. масса	Брутто-формула<br>$C_{20}H_{16}Cl_2N_2O_2S$
	Физ. состояние<br>***порошок***	Т. пл. (Т. кип.)<br>181-182°C	Устойчивость при хранении<br>***устойчив***

Синтетик (лаборатория, факультет, подпись, телефон)<br>**Милутка М.**С. Отдел координационных соединений НИИФОХ ЮФУ<br>+7(863) 297-51-89	Зав. лаб. (рук. гр.) Фамилия, подпись<br>**А.С. Бурлов,**
Оператор (Фамилия, подпись)<br>Этметченко	Комментарии оператора<br>***нет***

ЯМР	ИК	УФ	Элементный анализ				
Исследуемое ядро			Элемент	C	H	N	Др.
Растворитель			Вычислено, %	57.29	3.85	6.68	
Стандарт			Найдено, %	57,23<br>57,27	3,85<br>3,93	6,60<br>6,64	

ср. 57,25 3,89 6,62

БЛАНК ЗАКАЗА НА ПРОВЕДЕНИЕ ИССЛЕДОВАНИЯ СТРУКТУРЫ СОЕДИНЕНИЙ

1d

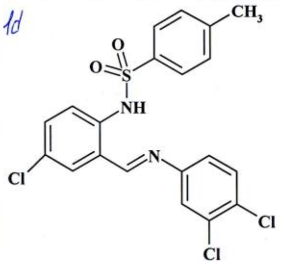

	Дата получения заказа<br>09.03.2020	Дата проведения анализа<br>16.03.2020	Масса образца, мг
	Шифр<br>**М-148**	Мол. масса	Брутто-формула<br>$C_{20}H_{15}Cl_3N_2O_2S$
	Физ. состояние<br>***порошок***	Т. пл. (Т. кип.)<br>154-155°C	Устойчивость при хранении<br>***устойчив***

Синтетик (лаборатория, факультет, подпись, телефон)<br>**Милутка М.**С. Отдел координационных соединений НИИФОХ ЮФУ<br>+7(863) 297-51-89	Зав. лаб. (рук. гр.) Фамилия, подпись<br>**А.С. Бурлов,**
Оператор (Фамилия, подпись)<br>Этметченко	Комментарии оператора<br>***нет***

ЯМР	ИК	УФ	Элементный анализ				
Исследуемое ядро			Элемент	C	H	N	Др.
Растворитель			Вычислено, %	52.94	3.33	6.17	
Стандарт			Найдено, %	52,87<br>52,93	3,33<br>3,41	6,17<br>6,25	

ср. 52,90 3,37 6,21

БЛАНК ЗАКАЗА НА ПРОВЕДЕНИЕ ИССЛЕДОВАНИЯ СТРУКТУРЫ СОЕДИНЕНИЙ

1e

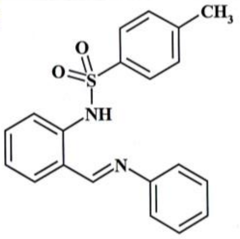

Дата получения заказа<br>11.03.2020	Дата проведения анализа<br>18.03.2020	Масса образца, мг
Шифр<br>**B962**	Мол. масса	Брутто-формула<br>$C_{20}H_{18}N_2O_2S$
Физ. состояние<br>***порошок***	Т. пл. (Т. кип.)<br>99-100˚С	Устойчивость при хранении<br>***устойчив***

Синтетик (лаборатория, факультет, подпись, телефон)<br>**Милутка М.**С. Отдел координационных соединений НИИФОХ ЮФУ<br>+7(863) 297-51-89	Зав. лаб. (рук. гр.) Фамилия, подпись<br>**А.С. Бурлов**,
Оператор (Фамилия, подпись)<br>Этметченко	Комментарии оператора<br>***нет***

ЯМР	ИК	УФ	Элементный анализ				
Исследуемое ядро			Элемент	C	H	N	Др.
Растворитель			Вычислено, %	68.55	5.18	7.99	
Стандарт			Найдено, %	68,48<br>68,54	5,04<br>5,13	7,87<br>7,95	

ср. 68,51 5,09 7,91

БЛАНК ЗАКАЗА НА ПРОВЕДЕНИЕ ИССЛЕДОВАНИЯ СТРУКТУРЫ СОЕДИНЕНИЙ

2a

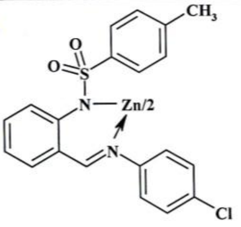

Дата получения заказа<br>03.08.2020	Дата проведения анализа<br>10.08.2020	Масса образца, мг
Шифр<br>**М-154**	Мол. масса	Брутто-формула<br>$C_{40}H_{32}Cl_2N_4O_4S_2Zn$
Физ. состояние<br>***порошок***	Т. пл. (Т. кип.)<br>>300˚С	Устойчивость при хранении<br>***устойчив***

Синтетик (лаборатория, факультет, подпись, телефон)<br>**Милутка М.**С. Отдел координационных соединений НИИФОХ ЮФУ<br>+7(863) 297-51-89	Зав. лаб. (рук. гр.) Фамилия, подпись<br>**А.С. Бурлов**,
Оператор (Фамилия, подпись)<br>Этметченко	Комментарии оператора<br>***нет***

ЯМР	ИК	УФ	Элементный анализ				
Исследуемое ядро			Элемент	C	H	N	Др.
Растворитель			Вычислено, %	57.67	3.87	6.72	
Стандарт			Найдено, %	57,59<br>57,65	3,87<br>3,93	6,67<br>6,73	

ср. 57,62 3,90 6,70

БЛАНК ЗАКАЗА НА ПРОВЕДЕНИЕ ИССЛЕДОВАНИЯ СТРУКТУРЫ СОЕДИНЕНИЙ

2b

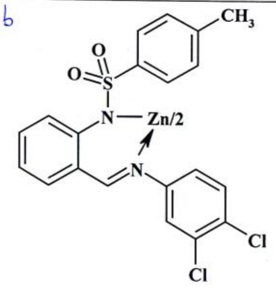

Дата получения заказа	Дата проведения анализа	Масса образца, мг
05.08.2020	12.08.2020	
Шифр<br>**М-156**	Мол. масса	Брутто-формула<br>$C_{40}H_{30}Cl_4N_4O_4S_2Zn$
Физ. состояние<br>***порошок***	Т. пл. (Т. кип.)<br>>300°C	Устойчивость при хранении<br>***устойчив***

Синтетик (лаборатория, факультет, подпись, телефон)	Зав. лаб. (рук. гр.) Фамилия, подпись
**Милутка М.**С. Отдел координационных соединений НИИФОХ ЮФУ<br>+7(863) 297-51-89 [signature]	**А.С. Бурлов,** [signature]
Оператор (Фамилия, подпись)<br>Этметченко	Комментарии оператора<br>***нет***

ЯМР	ИК	УФ	Элементный анализ				
Исследуемое ядро			Элемент	C	H	N	Др.
Растворитель			Вычислено, %	53.26	3.35	6.21	
Стандарт			Найдено, %	53,19<br>53,23	3,37<br>3,41	6,21<br>6,25	

ср. 53,21 3,39 6,23

БЛАНК ЗАКАЗА НА ПРОВЕДЕНИЕ ИССЛЕДОВАНИЯ СТРУКТУРЫ СОЕДИНЕНИЙ

2c

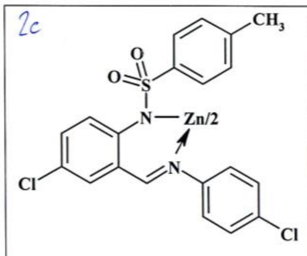

Дата получения заказа	Дата проведения анализа	Масса образца, мг
27.08.2020	03.09.2020	
Шифр<br>**М-147**	Мол. масса	Брутто-формула<br>$C_{40}H_{30}Cl_4N_4O_4S_2Zn$
Физ. состояние<br>***порошок***	Т. пл. (Т. кип.)<br>>300°C	Устойчивость при хранении<br>***устойчив***

Синтетик (лаборатория, факультет, подпись, телефон)	Зав. лаб. (рук. гр.) Фамилия, подпись
**Милутка М.**С. Отдел координационных соединений НИИФОХ ЮФУ<br>+7(863) 297-51-89 [signature]	**А.С. Бурлов,** [signature]
Оператор (Фамилия, подпись)<br>Этметченко	Комментарии оператора<br>***нет***

ЯМР	ИК	УФ	Элементный анализ				
Исследуемое ядро			Элемент	C	H	N	Др.
Растворитель			Вычислено, %	53.26	3.35	6.21	
Стандарт			Найдено, %	53,18<br>53,26	3,35<br>3,43	6,21<br>6,29	

ср. 53,22 3,39 6,25

БЛАНК ЗАКАЗА НА ПРОВЕДЕНИЕ ИССЛЕДОВАНИЯ СТРУКТУРЫ СОЕДИНЕНИЙ

2d 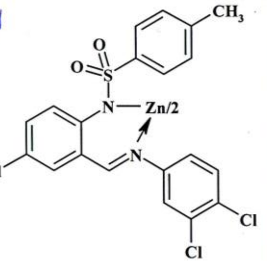	Дата получения заказа<br>31.08.2020	Дата проведения анализа<br>07.09.2020	Масса образца, мг
	Шифр<br>**М-152**	Мол. масса	Брутто-формула<br>$C_{40}H_{28}Cl_6N_4O_4S_2Zn$
	Физ. состояние<br>***порошок***	Т. пл. (Т. кип.)<br>273-274˚С	Устойчивость при хранении<br>***устойчив***

Синтетик (лаборатория, факультет, подпись, телефон)<br>**Милутка М.**С. Отдел координационных соединений НИИФОХ ЮФУ<br>+7(863) 297-51-89	Зав. лаб. (рук. гр.) Фамилия, подпись<br>**А.С. Бурлов,**
Оператор (Фамилия, подпись)<br>Этметченко	Комментарии оператора<br>***нет***

ЯМР	ИК	УФ	Элементный анализ				
Исследуемое ядро			Элемент	**C**	**H**	**N**	Др.
Растворитель			Вычислено, %	49.48	2.91	5.77	
Стандарт			Найдено, %	49,40<br>49,46	2,92<br>2,98	5,73<br>5,77	
			ср.	49,43	2,95	5,75	

БЛАНК ЗАКАЗА НА ПРОВЕДЕНИЕ ИССЛЕДОВАНИЯ СТРУКТУРЫ СОЕДИНЕНИЙ

2e 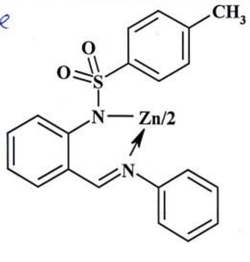	Дата получения заказа<br>03.09.2020	Дата проведения анализа<br>10.09.2020	Масса образца, мг
	Шифр<br>**В947**	Мол. масса	Брутто-формула<br>$C_{40}H_{34}N_4O_4S_2Zn$
	Физ. состояние<br>***порошок***	Т. пл. (Т. кип.)<br>295-296˚С	Устойчивость при хранении<br>***устойчив***

Синтетик (лаборатория, факультет, подпись, телефон)<br>**Милутка М.**С. Отдел координационных соединений НИИФОХ ЮФУ<br>+7(863) 297-51-89	Зав. лаб. (рук. гр.) Фамилия, подпись<br>**А.С. Бурлов,**
Оператор (Фамилия, подпись)<br>Этметченко	Комментарии оператора<br>***нет***

ЯМР	ИК	УФ	Элементный анализ				
Исследуемое ядро			Элемент	**C**	**H**	**N**	Др.
Растворитель			Вычислено, %	62.86	4.48	7.33	
Стандарт			Найдено, %	62,67<br>62,71	4,50<br>4,54	7,38<br>7,42	
			ср.	62,69	4,52	7,40	

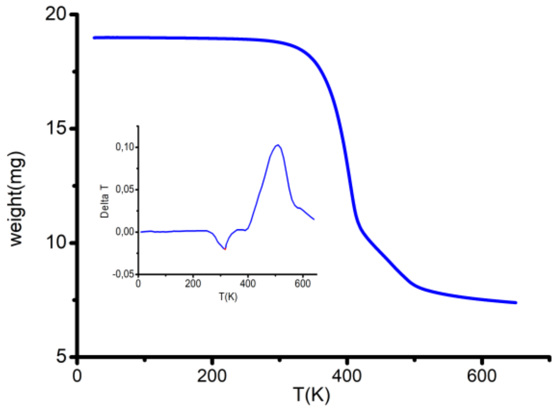
weight(mg)
T(K)
Delta T
T(K)

a

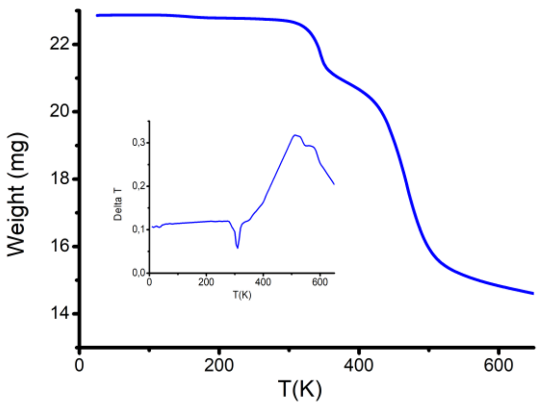
Weight (mg)
T(K)
Delta T
T(K)

b

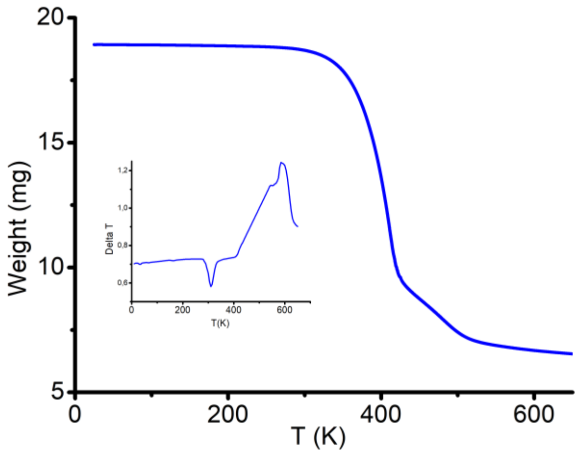
Weight (mg)
T (K)
Delta T
T(K)

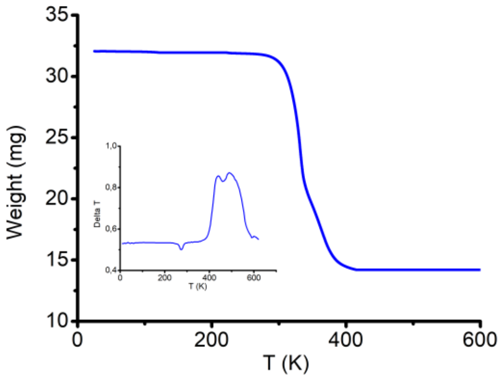
Weight (mg)
T (K)
Delta T
T (K)
